# Qualitative and quantitative analysis of flavonoids from 12 species of Korean mulberry leaves

**DOI:** 10.1007/s13197-018-3093-2

**Published:** 2018-03-19

**Authors:** Wan-Taek Ju, O-Chul Kwon, Hyun-Bok Kim, Gyoo-Byung Sung, Heon-Woong Kim, Yong-Soon Kim

**Affiliations:** National Academy of Agricultural Science, RDA, Wanju-gun, 565-851 Republic of Korea

**Keywords:** *Morus alba* L., Mulberry leaves, Flavonoids, UPLC–DAD–QTOF/MS

## Abstract

The total flavonoids in leaves of 12 varieties of Korean mulberry (*Morus alba* L.) were determined. Seventeen flavonoids were isolated and analyzed using ultra-performance liquid chromatography coupled with diode array detection and quadrupole time-of-flight mass spectrometry (UPLC–DAD–QTOF/MS). To determine the flavonoid contents, HPLC analysis was performed on these 17 flavonoids. The total flavonoid contents of the 12 varieties of mulberry leaves ranged from 748.5 to 1297.9 mg, with the highest obtained from the Cheong Su variety (1297.9 ± 112.0 mg). Among the 17 flavonoids analyzed, quercetin 3-*O*-rutinoside (rutin) and quercetin 3-*O*-glucoside (isoquercitrin) had highest contents in the Cheong Su variety. Furthermore, the Dae Dang Sang variety gave the highest quercetin 3-*O*-rutinoside (rutin) content among the mulberry leaves investigated, at 425.5 ± 45.9 mg. Major flavonols from Dae Dang Sang were detected by UPLC–DAD–QTOF/MS. A total of 17 flavonoid compound peaks were identified in the analysis time range of 5–40 min, all of which were kaempferol and quercetin glycosides. Seven of the 17 compounds identified in mulberry leaves were unknown.

## Introduction

Mulberry (*Morus* spp.) is a deciduous tree that belongs to the genus *Morus* from the Moraceae family that consists of 10–16 species and is widely distributed in tropical, subtropical, and temperate regions globally (Jeong et al. 2014). Mulberry has been used in East Asia (Korea, China, and Japan) as an herbal medicine due to its various pharmacological effects, including antihyperglycemic (Singab et al. 2005), antiallergic (Chai et al. 2005), and immunomodulatory activities (Bharani et al. 2010).

Mulberry has been recognized as a potentially important functional food due to its biologically active compounds, which include flavonoids (anthocyanin, rutin, quercetin, and isoquercitrin), steroids, amino acids, polysaccharides, γ-aminobutyric acid (GABA), vitamins, and 1-deoxynojirimycin (DNJ) (Kim et al. 2003a, b; Choi and Hwang 2005; Wang et al. 2008; Zhang et al. 2016). In Korea and Japan, mulberry leaves are consumed as antihyperglycemic nutraceutical foods by diabetic patients (Kim et al. 2003a, b).

Flavonoids are a large group of polyphenolic compounds found in fruits, vegetables, and herbs (Enkhmaa et al. 2005). Plants of the genus *Morus* are known to be rich in flavonoids, including quercetin 3-(6-malonylglucoside), rutin, isoquercitin (Katsube et al. 2006), cyanidin 3-rutinoside, and cyanidin 3-glucoside (Chen et al. 2006). These compounds are known to have potential antioxidant properties and probable roles in preventing oxidative stress-associated diseases (Haminiuk et al. 2012). Several researchers have studied the isolation, identification, and contents of flavonoid components in various mulberry species. Lee et al. (2004) reported that the five flavonoid contents for 20 cultivars of mulberry fruits varied from 9.80 to 69.69 mg/100 g (dry weight) through quantitative analysis with high performance liquid chromatography (HPLC). Furthermore, Katsube et al. (2006) identified that quercetin 3-(6-malonylglucoside) was the most abundant active component in dried mulberry leaves. Recently, Thabti et al. (2012) reported the identification and quantification of phenolic acids and flavonol glycosides in leaves of three mulberry species (*M. alba* var. *alba*, *M. alba* var. *rosa*, and *M. rubra*) using HPLC–DAD and HPLC–MS. They also reported the first identification of kaempferol-7-*O*-glucoside, quercetin-3-*O*-β-glucoside-7-*O*-α-rhamnoside, and quercetin-3-*O*-rhamnoside-7-*O*-glucoside in mulberry leaves. Furthermore, Thabti et al. (2014) reported that the highest total flavonoid content among leaves of three mulberry species were detected in *M. rubra*. In Korea, flavonoids in various species of mulberry leaves have not been investigated and their compositions are unknown. Furthermore, the utilization of different *Morus* species has been attempted, with interspecific hybridization conducted to incorporate desirable characteristics for crop improvement. Improving the foliage characteristics of mulberry (*Morus* spp.), both quantitatively and qualitatively, is the long-term goal for mulberry breeders. Therefore, in this work, we have determined the flavonoid contents of leaves from 12 varieties of mulberry using ultra-performance liquid chromatography coupled with diode array detection and quadrupole time-of-flight mass spectrometry (UPLC–DAD–QTOF/MS). This study aimed to provide basic mulberry breeding information for commercial purposes and functional utilization.

## Materials and methods

### Plant material and reagents

Leaves from 12 mulberry species were collected from the Sericulture and Apiculture Division of the Department of Agricultural Biology, RDA (Jeon-Ju, South Korea). All mulberry leaf samples were cleaned and dried in a lyophilizer. All dried samples were pulverized and stored below – 18 °C prior to analysis. HPLC-grade acetonitrile, methanol, and water were obtained from Fisher Scientific (Fair Lawn, NJ, USA). Formic acid was purchased from Junsei Chemical (Tokyo, Japan). Galangin (Sigma Aldrich Co., St. Louis, MO, USA) was used as the internal standard solution.

### Preparation of samples for instrument analysis

Sample extraction was conducted according to the method described by Kim et al. (2012) with minor modifications. The powdered leave (1 g) was mixed with 10 mL of acidified hydroalcoholic solvent (methanol/water/formic acid = 50:45:5, v/v/v) containing galangin (100 ppm) as internal standard. To extract flavonoids, the mixture was vortexed, stirred with a shaker (5 min, 200 rpm, and room temperature), and then centrifuged for 15 min at 3000 rpm and 10 °C. The supernatant was filtered using a syringe filter (0.45 μm, PTFE, Whatman, Kent, England). A 0.5-mL aliquot of the filtrate was then diluted with water to a final volume of 5 mL. The flavonoid extract was then purified and isolated by solid-phase extraction using Sep-Pak C-18 cartridge (Waters Co., Milford, MA, USA). Sep-Pak activation was performed by washing the cartridge with methanol (2 mL), followed by water (2 mL) for conditioning. The diluted extract was then loaded onto the cartridge and impurities were removed by washing with water (2 mL). Finally, the flavonoid mixture was eluted using methanol (3 mL). The purified extract was then concentrated by blowing with N_2_ gas, and then redissolved in the extraction solvent (0.5 mL) without internal standard prior to instrument analysis. All experimental analyses were performed in triplicate.

### Instrumentation

Ultra-performance liquid chromatography (UPLC) with photo diode array detector, set at 280 and 320 nm, coupled with quadrupole time-of-flight mass spectroscopy (UPLC–DAD–QTOF/MS; Waters Co., Milford, MA, USA) was used for analysis. UV spectra were measured in the region of 210–600 nm. Chromatographic conditions were as follows: Column, Luna Omega 1.6 μm C18 (150 × 2.1 mm, Phenomenex); precolumn: SecurityGuard ULTRA cartridge (UHPLC C18 for 2.1 ID column, Phenomenex); column temperature, 30 °C; mobile phase, 0.5% formic acid in water (A) and 0.5% formic acid in acetonitrile (B); flow rate, 0.3 mL/min; injection volume, 5 μL; total running time, 60 min; and gradient elution profile: 0–2 min, 7% B; 24 min, 15% B; 40 min, 30% B; 48–50 min, 60% B; 53–54 min, 90% B; 55–60 min, 7% B. Mass analysis conditions were as follows: ion source temperature, 120 °C; desolvation temperature, 400 °C; desolvation gas, 1000 L/h; cone gas, 30 L/h; capillary voltage, 3500 V; sampling cone voltage, 40 V; ion mode, positive; and mass range, *m/z* 50–800.

## Results and discussion

### Isolation and identification of flavonoids from mulberry leaves

Seventeen flavonoids were isolated from mulberry leaves and analyzed using UPLC–DAD–QTOF/MS (Table 1), as follows: quercetin 3-*O*-rutinoside-7-*O*-glucoside (morkotin A)^NFL^ (peak 1), quercetin 3,7-di-*O*-glucoside (peak 2), quercetin 3-*O*-rutinoside-7-*O*-rhamnoside (morkotin B)^NFL^ (peak 3), kaempferol 3-*O*-rutinoside-7-*O*-glucoside (moragrol A)^NFL^ (peak 4), quercetin 3-*O*-glucoside (isoquercitrin) (peak 5), kaempferol 3,7-di-*O*-glucoside (peak 6), kaempferol 3-*O*-rutinoside-7-*O*-rhamnoside (moragrol B)^NFL^ (peak 7), quercetin 3-*O*-rutinoside (rutin) (peak 8), kaempferol 3-*O*-rhamnoside-7-*O*-glucoside (peak 9), quercetin 3-*O*-glucoside (isoquercitrin) (peak 10), quercetin 3-*O*-(6″-O-malonyl)glucoside (peak 11), kaempferol 3-*O*-rutinoside (nicotiflorin) (peak 12), kaempferol 3-*O*-(6″-*O*-malonyl)glucoside-7-*O*-rhamnoside (moragrol C)^NFL^ (peak 13), kaempferol 3-*O*-glucoside (astragalin) (peak 14), quercetin 3-*O*-(2″-*O*-malonyl)glucoside (morkotin C)^NFL^ (peak 15), kaempferol 3-*O*-(6″-*O*-malonyl)glucoside (peak 16), and kaempferol 3-*O*-(2″-*O*-malonyl)glucoside (moragrol D)^NFL^ (peak 17) (NFL = new flavonoid in mulberry leaves). Most kaempferol and quercetin in mulberry leaves naturally existed as glycosides (Thabti et al. 2012). Another study by Katsube et al. (2006) identified quercetin-3-*O*-(6-*O*-malonyl)-β-d-glucoside (QMG) and kaempferol-3-*O*-(6-*O*-malonyl)-β-d-glucoside (KMG) from mulberry leaves, with QMG found to be more abundant. Therefore, no information is available on the isolation and identification of these 17 flavonoids from varieties of mulberry leaves produced in Korea.

**Table 1:** Seventeen flavonoids isolated from leaves of mulberry (*Morus alba* L.) and their mass spectrometry data

Aglycones	Glycosides	Acylations	Peak no.	Individual flavonols	MW	Fragment ions (*m/z*)
Kaempferol (*m/z* 287)	Mono		14	Kaempferol 3-*O*-glucoside (astragalin)	448	471, 449, 287
		Mal	16	Kaempferol 3-*O*-(6″-*O*-malonyl)glucoside	534	557, 535, 287
		Mal	17	Kaempferol 3-*O*-(2″-*O*-malonyl)glucoside (moragrol D)^NFL^	534	557, 535, 287
	Di		9	Kaempferol 3-*O*-rhamnoside-7-*O*-glucoside	594	617, 595, 449, 287
			12	Kaempferol 3-*O*-rutinoside (nicotiflorin)	594	617, 595, 449, 287
			6	Kaempferol 3,7-di-*O*-glucoside	610	633, 611, 449, 287
		Mal	13	Kaempferol 3-*O*-(6″-*O*-malonyl)glucoside-7-*O*-rhamnoside (moragrol C)^NFL^	680	703, 681, 535, 433, 287
	Tri		7	Kaempferol 3-*O*-rutinoside-7-*O*-rhamnoside (moragrol B)^NFL^	740	763, 741, 595, 449, 433, 287
			4	Kaempferol 3-*O*-rutinoside-7-*O*-glucoside (moragrol A)^NFL^	756	779, 757, 611, 595, 449, 287
Quercetin (*m/z* 303)	Mono		10	Quercetin 3-*O*-glucoside (isoquercitrin)	464	487, 465, 303
		Mal	11	Quercetin 3-*O*-(6″-*O*-malonyl)glucoside	550	573, 551, 465, 303
			15	Quercetin 3-*O*-(2″-*O*-malonyl)glucoside (morkotin C)^NFL^	550	573, 551, 303
	Di		5	Quercetin 3-*O*-rhamnoside-7-*O*-glucoside	610	633, 611, 465, 303
			8	Quercetin 3-*O*-rutinoside (rutin)	610	633, 611, 465, 449, 303
			2	Quercetin 3,7-di-*O*-glucoside	626	649, 627, 465, 303
	Tri		3	Quercetin 3-*O*-rutinoside-7-*O*-rhamnoside (morkotin B)^NFL^	756	779, 757, 611, 465, 449, 303
			1	Quercetin 3-*O*-rutinoside-7-*O*-glucoside (morkotin A)^NFL^	772	795, 773, 627, 611, 465, 303

*NFL* new flavonoid in mulberry leaves

All samples analyzed in positive ion mode (*m/z*, [M+H]^+^) using UPLC–DAD–QTOF/MS

Each value calculated as mean ± SD of three replicates using internal standard (galangin)

### Quantification of flavonoids in mulberry leaves

**Fig. 1:**
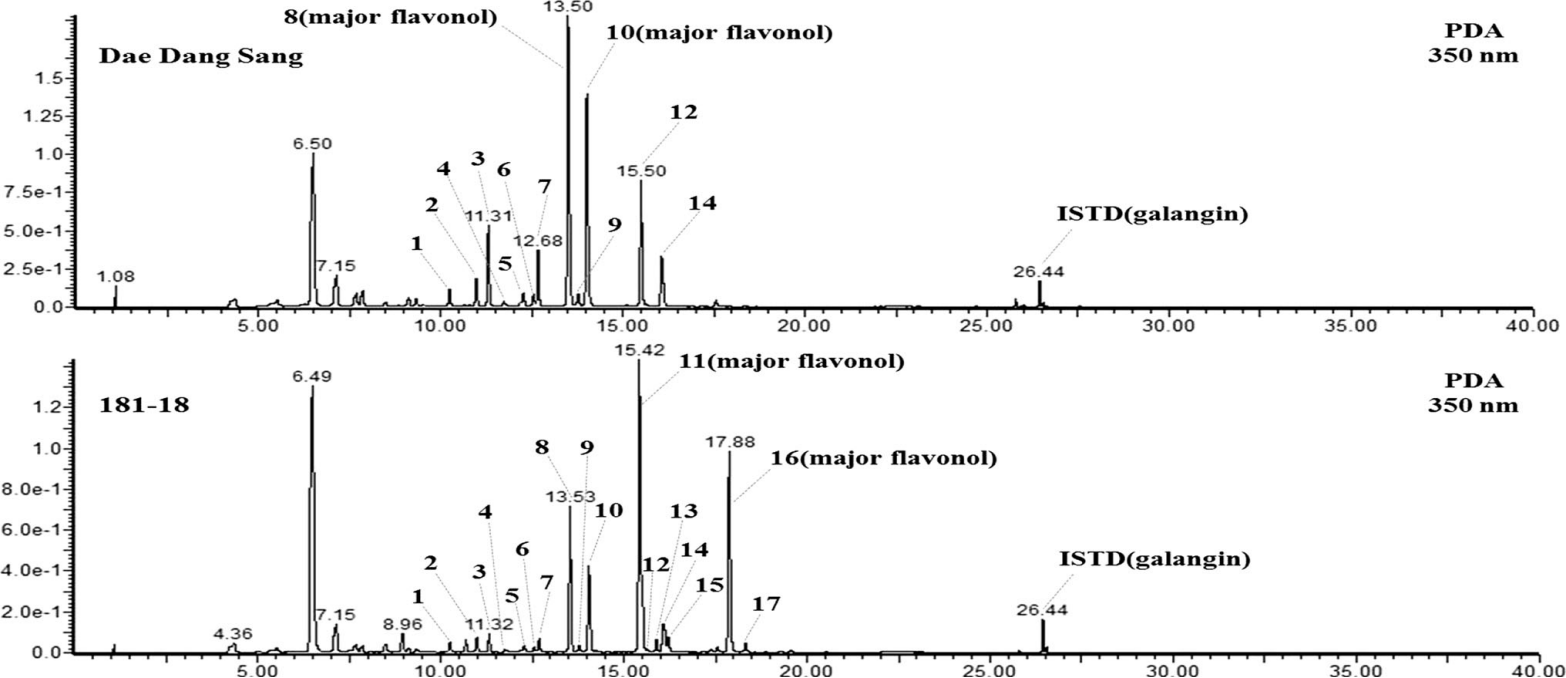
LC chromatograms of flavonoids in Dae Dang Sang and 181-18 isolated from Korean mulberry leaves (*Morus alba* L.)

To determine the flavonoid contents in 12 varieties of mulberry leaves, HPLC was performed using the 17 flavonoids isolated from the mulberry leaves. As shown in Table 2, the total flavonoid contents ranged from 748.5 to 1297.9 mg for the 12 varieties of mulberry leaves. The variety with the highest total flavonoid content was Cheong Su (1297.9 ± 112.0 mg). For comparison, Thabti et al. (2012) reported that the total flavonoid content of *Morus rubra* ranged from 193.87 to 398.33 mg RE/100 g DW, and was quantified as 450 mg (aqueous extracts) for the stem bark of *M. alba* var. *alba*. In Cheong Su, quercetin 3-*O*-rutinoside (rutin) and quercetin 3-*O*-glucoside (isoquercitrin) had the highest contents among the 17 flavonoids analyzed. Furthermore, in Dae Dang Sang, the quercetin 3-*O*-glucoside (isoquercitrin) content was lower than that of Cheong Su, while the quercetin 3-*O*-rutinoside (rutin) content was the highest among the mulberry leaves investigated, at 425.5 ± 45.9 mg. Major compounds (quercetin 3-*O*-rutinoside (rutin) and quercetin 3-*O*-glucoside (isoquercitrin)) were detected at retention times of 13.5 and 14.01 min in the LC chromatogram (peaks 8 and 10) from Dae Dang Sang (Fig. 1). Both Cheong Su and Dae Dang Sang had similar flavonoid contents. Buckwheat and some plant leaves have been shown to have rutin contents of 4–9% dry weight depending upon the stage of plant development (Kalinova et al. 2006). For dry fruits and vegetables, the rutin content was found to show little variation, ranging from 0.15 to 0.18% dry weight (Kalinova et al. 2006). Furthermore, isoquercitrin is a natural flavonoid glucoside found in medicinal and dietary plants, such as vegetables, herbs, and flowers, and, together with rutin, is a major glycosidic form of natural flavonol quercetin. Another study reported the isolation of isoquercitrin from *Annona squamosa* and demonstrated its protective action on diabetes mellitus, which is possibly mediated by enhanced insulin synthesis/secretion and/or decreased glucose-6-phosphatase activity (Panda and Kar 2007). From these results, we concluded that Cheong Su and Dae Dang Sang were good varieties for breeding and functional food development.

**Table 2:** Contents (mg/100 g DW) of 17 flavonoids isolated from mulberry leaves (*Morus alba* L.)

Peak no.	Cheong-Il^(1)^	Hwan ship Jo Saeng^(2)^	Su Hyang^(3)^	Dae Shim^(4)^	Cheong-Il 4X^(5)^	180-11^(6)^	Shim Heung^(7)^	Cheong Su^(8)^	180-12^(9)^	Dae Dang Sang^(10)^	Baek Ok Wang^(11)^	181-18^(12)^
1	8.8 ± 0.7	10.3 ± 1.0	10.6 ± 0.9	6.7 ± 0.3	11.8 ± 1.1	10.6 ± 1.1	4.9 ± 0.4	18.7 ± 1.5	4.6 ± 0.3	23.9 ± 2.4	3.5 ± 0.3	10.9 ± 0.7
2	14.3 ± 1.2	14.1 ± 1.5	17.3 ± 1.7	12.3 ± 0.5	19.1 ± 1.9	11.4 ± 0.9	8.5 ± 0.6	49.0 ± 3.9	10.9 ± 0.3	37.1 ± 3.7	7.0 ± 0.6	15.3 ± 1.2
3	10.5 ± 0.3	7.7 ± 1.0	6.8 ± 0.8	17.4 ± 0.6	8.1 ± 0.8	44.6 ± 4.0	20.8 ± 1.3	62.8 ± 5.4	21.6 ± 0.9	95.0 ± 10.6	22.0 ± 1.8	17.2 ± 1.1
4	1.5 ± 0.1	2.2 ± 0.2	6.6 ± 1.9	4.2 ± 1.2	9.1 ± 1.0	3.7 ± 0.8	1.2 ± 0.1	5.7 ± 0.5	2.5 ± 0.2	7.3 ± 1.0	ND	2.4 ± 0.2
5	7.0 ± 1.0	5.2 ± 0.6	3.6 ± 0.6	8.1 ± 1.0	5.1 ± 0.9	12.0 ± 2.0	11.9 ± 2.5	33.9 ± 2.9	9.4 ± 1.3	20.4 ± 3.7	14.6 ± 2.9	7.5 ± 1.0
6	3.8 ± 0.5	2.7 ± 0.4	4.5 ± 0.3	5.3 ± 0.3	3.5 ± 0.4	3.0 ± 0.2	3.6 ± 0.7	24.9 ± 2.2	5.4 ± 0.7	16.3 ± 1.6	ND	5.6 ± 0.2
7	5.2 ± 0.5	4.2 ± 0.0	5.4 ± 0.4	10.2 ± 0.2	6.3 ± 0.5	21.0 ± 1.4	9.6 ± 0.6	43.3 ± 3.7	13.6 ± 0.7	64.6 ± 7.0	12.1 ± 1.0	12.4 ± 1.1
8	136.6 ± 12.3	119.5 ± 13.2	139.5 ± 12.7	146.7 ± 7.0	166.1 ± 15.3	245.6 ± 21.0	90.0 ± 7.4	375.9 ± 32.3	233.2 ± 9.8	425.5 ± 45.9	103.8 ± 10.2	136.0 ± 3.5
9	3.2 ± 0.4	2.4 ± 0.2	ND	3.5 ± 0.2	1.3 ± 0.2	5.8 ± 0.6	6.6 ± 1.1	32.4 ± 2.8	6.2 ± 0.3	16.7 ± 1.8	7.0 ± 0.9	7.0 ± 0.7
10	137.9 ± 17.8	85.9 ± 12.6	92.9 ± 14.9	123.2 ± 10.9	142.4 ± 20.3	100.4 ± 13.5	75.4 ± 27.0	369.8 ± 32.6	155.4 ± 13.3	296.6 ± 31.5	89.0 ± 14.1	108.9 ± 12.4
11	283.9 ± 28.8	255.2 ± 26.3	312.5 ± 24.9	296.1 ± 9.0	361.4 ± 29.1	326.3 ± 23.0	228.9 ± 20.9	ND	219.0 ± 4.0	ND	235.3 ± 10.7	229.9 ± 12.8
12	166.4 ± 16.9	66.8 ± 6.9	103.2 ± 8.2	88.4 ± 2.7	167.9 ± 13.5	127.5 ± 9.0	93.0 ± 8.5	147.6 ± 12.8	182.3 ± 3.3	164.2 ± 18.1	108.1 ± 4.9	96.6 ± 5.4
13	8.9 ± 0.4	4.5 ± 0.6	4.2 ± 0.6	8.1 ± 0.4	5.0 ± 0.4	15.0 ± 1.1	11.9 ± 1.1	0.5 ± 0.1	9.8 ± 0.2	ND	11.6 ± 0.1	11.9 ± 0.7
14	38.6 ± 10.0	17.9 ± 3.4	75.1 ± 10.4	26.6 ± 3.5	31.9 ± 8.9	20.7 ± 1.8	62.9 ± 10.9	133.3 ± 11.5	33.6 ± 4.7	71.0 ± 7.4	25.0 ± 9.9	31.3 ± 5.9
15	21.5 ± 1.0	15.7 ± 1.3	20.8 ± 0.8	20.6 ± 0.5	25.3 ± 2.0	22.8 ± 2.5	16.2 ± 1.4	ND	20.5 ± 0.5	ND	16.6 ± 0.6	16.7 ± 1.6
16	201.2 ± 15.1	128.5 ± 11.4	202.7 ± 15.5	154.7 ± 4.9	217.4 ± 16.6	172.0 ± 12.1	130.8 ± 7.8	ND	204.0 ± 3.9	ND	132.3 ± 5.8	219.2 ± 13.4
17	8.7 ± 0.3	5.6 ± 0.4	8.7 ± 0.2	7.0 ± 0.1	8.6 ± 0.6	7.4 ± 0.8	5.9 ± 0.6	ND	8.8 ± 0.0	ND	5.4 ± 0.4	9.8 ± 0.8
Total	1058.0 ± 102.8	748.5 ± 79.3	1014.4 ± 93.1	939.3 ± 41.7	1190.1 ± 106.3	1149.5 ± 93.8	782.1 ± 66.5	1297.9 ± 112.0	1140.8 ± 43.1	1238.7 ± 133.2	812.7 ± 88.2	938.5 ± 60.1

Peak 1—quercetin 3-*O*-rutinoside-7-*O*-glucoside (morkotin A)^NFL^; peak 2—quercetin 3,7-di-*O*-glucoside; peak 3—quercetin 3-*O*-rutinoside-7-*O*-rhamnoside (morkotin B)^NFL^; peak 4—kaempferol 3-*O*-rutinoside-7-*O*-glucoside (moragrol A)^NFL^; peak 5—quercetin 3-*O*-rhamnoside-7-*O*-glucoside; peak 6—kaempferol 3,7-di-*O*-glucoside; peak 7—kaempferol 3-*O*-rutinoside-7-*O*-rhamnoside (moragrol B)^NFL^; peak 8—quercetin 3-*O*-rutinoside (rutin); peak 9—kaempferol 3-*O*-rhamnoside-7-*O*-glucoside; and peak 10—quercetin 3-*O*-glucoside (isoquercitrin); peak 11—quercetin 3-*O*-(6″-*O*-malonyl)glucoside; peak 12—kaempferol 3-*O*-rutinoside (nicotiflorin); peak 13—kaempferol 3-*O*-(6″-*O*-malonyl)glucoside-7-*O*-rhamnoside (moragrol C)^NFL^; peak 14—kaempferol 3-*O*-glucoside (astragalin); peak 15—quercetin 3-*O*-(2″-*O*-malonyl)glucoside (morkotin C)^NFL^; peak 16—kaempferol 3-*O*-(6″-*O*-malonyl)glucoside and peak 17—kaempferol 3-*O*-(2″-*O*-malonyl)glucoside (moragrol D)^NFL^. All samples were analyzed in positive ion mode (m/z, [M+H]+) using UPLC–DAD–QTOF/MS; Each value calculated as mean ± SD of three replicates using internal standard (galangin); NFL, new flavonoid identified in mulberry leaves; ND, not detected

In contrast, from Cheong-Il 4X, polyphenol compound quercetin 3-*O*-(6″-*O*-malonyl) glucoside was detected at 361.4 ± 29.1 mg. In a previous study, ethanol extraction was performed on Cheong-Il and quercetin 3-*O*-(6″-*O*-malonyl) glucoside, a main bioactive substance with antidiabetic and antiarteriosclerosis properties, was determined to have a content of 143.25 mg/100 g, while quantitative changes in the six polyphenols in Cheong-Il, which is a mulberry cultivar widely used as a material in mulberry leaf tea, were investigated for three different heat pre-treatments: steaming, roasting, and microwaving (Choi et al. 2015). Our data shows that the quercetin 3-*O*-(6″-*O*-malonyl) glucoside content of Cheong-Il was 283.9 ± 28.8 mg. The each other reason was due to other cultivation region and climate, even though same variety phenol compound. In contrast, quercetin 3-*O*-(6″-*O*-malonyl) glucoside was not detected on Cheong Su and Dae Dang Sang.

### HPLC–DAD–ESI/MS analysis

**Fig. 2:**
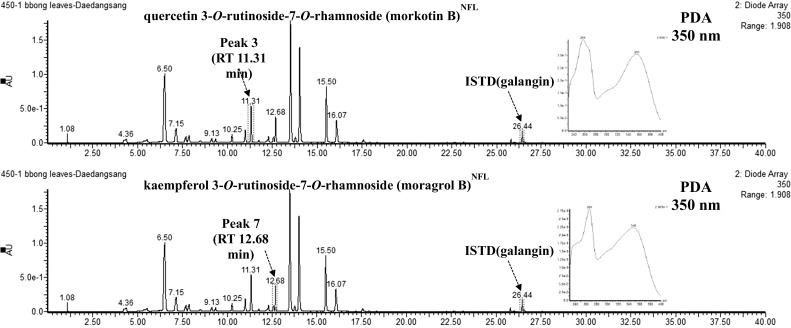
LC chromatograms and UV spectrums of new compounds (morkotin B, moragrol B) isolated from Korean mulberry leaves

To obtain the molecular mass of flavonoids detected by HPLC–DAD, HPLC–ESI–MS analysis of the fractionated extract was performed. All samples were analyzed in positive ion mode (*m/z*, [M+H]^+^) using UPLC–DAD–QTOF/MS. UPLC–DAD–QTOF/MS chromatograms of the major flavonols detected in Dae Dang Sang are shown in Fig. 2. Almost all flavonoids showed [M+Na]^+^ or [M+H]^+^ ions depending on the mass of the compound. A total of 17 peaks were identified as flavonoid compounds in the analysis time of 5–40 min, all of which were kaempferol and quercetin glycosides.

Peaks 1, 2, 3, 5, 8, 10, 11, and 15 corresponded to quercetin derivatives, as confirmed by [quercetin+H]^+^ ion peak by MS, while others (4, 6, 7, 9, 12, 13, 14, 16, and 17) were kaempferol derivatives, as confirmed by the [kaempferol+H]^+^ ion peak. The major peak of Dae Dang Sang (peak 8) generated MS fragments of *m/z* 633, 611, 465, 449, and 303, and was assigned as rutin (quercetin 3-*O*-rutinoside). This compound had already been identified in *M. alba* dried leaves using NMR and MS techniques (Katsube et al. 2006).

**Fig. 3:**
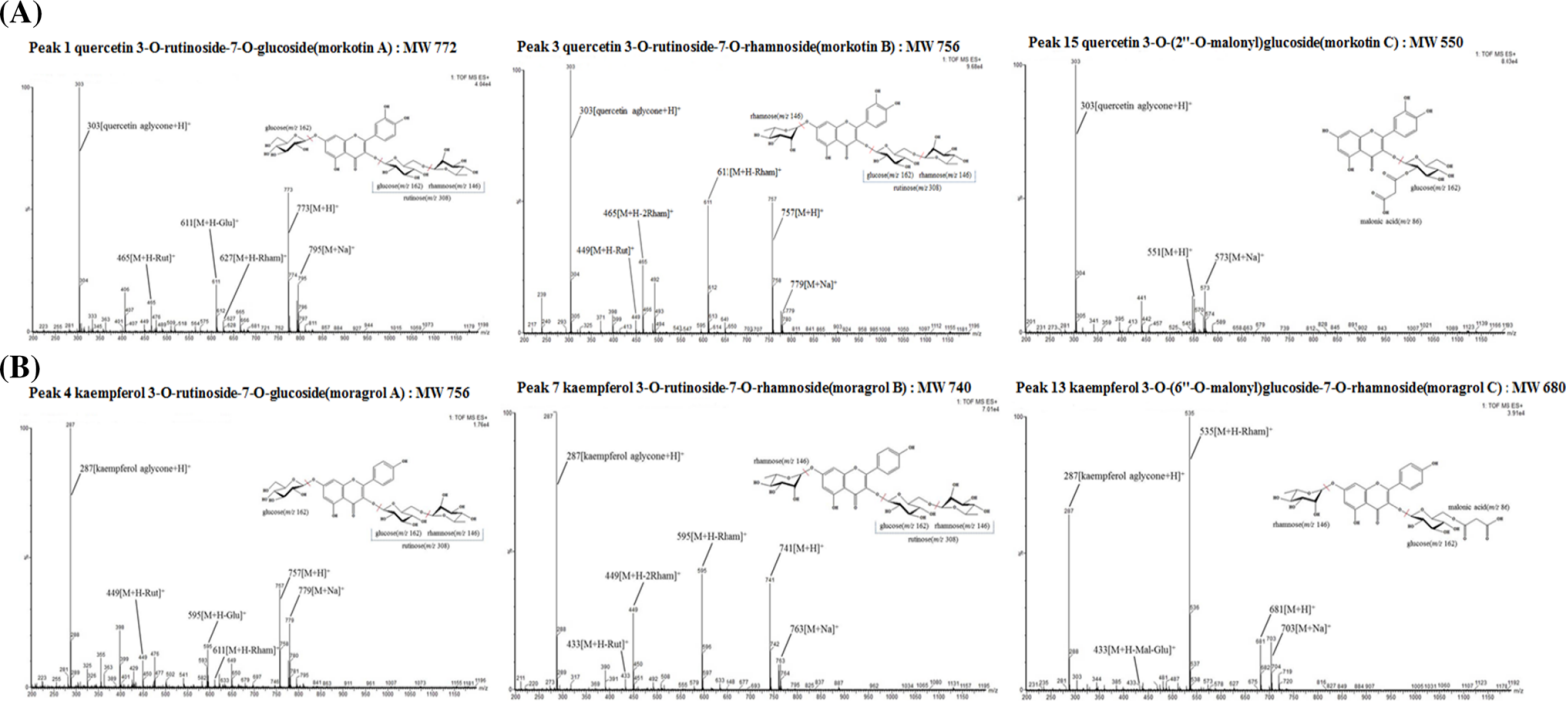
Mass spectra of new flavonoids detected from extracting mulberry leaves: **a** glycoside derivatives of quercetin, **b** glycoside derivatives of kaempferol

Glycosides 1, 3, and 15 of quercetin and 4, 7, 13, 17 of kaempferol have not previously been extracted from mulberry leaves. Compounds 1, 3, and 15 all showed an intense MS2 ion at *m/z* 303, suggesting that they were glycoside derivatives of quercetin. Fragment ion patterns of three new quercetin compounds from mulberry leaves corresponded to [M+Na]^+^, [M+H]^+^, [M+H-Rham]^+^, [M+H-Glu-Rham]^+^, and [M+H-2Glu-Rham]^+^ (Fig. 3a). Quercetin 3-*O*-rutinoside-7-*O*-rhamnoside had the highest content at 11.31 min. Quercetin *O*-glycosides are quercetin derivatives with at least one *O*-glycosidic bond, and are widely distributed in plants. Quercetin 3-*O*-glycosides can occur as monosaccharides with glucose, galactose, rhamnose, or xylose. These compounds are found in various fruits, vegetables, and other anatomical parts of plants (Wiczkowski and Piskuła 2004). Quercetin 3-*O*-glucoside has been found, among others, in mango fruit (Berardini et al. 2005), whereas quercetin 3-*O*-rhamnoside has been detected in spinach (Kuti and Konuru 2004) and peppers (Materska et al. 2003). Quercetin 3-*O*-β-glucoside-7-*O*-α-rhamnoside has been isolated from leaves of *Cotoneaster* species (Kicel et al. 2016) and some Italian *Aconitum* species (Braca et al. 2003). In contrast, compounds 4, 7, 13, and 17 all showed an intense MS2 ion at *m/z* 287, suggesting that they were glycoside derivatives of kaempferol. Fragment ion patterns of these four new kaempferol compounds corresponded to [M+Na]^+^, [M+H]^+^, [M+H-Rham]^+^, [M+H-Glu-Rham]^+^, and [M+H-2Glu-Rham]^+^ (Fig. 3b). Kaempferol 3-*O*-rutinoside-7-*O*-rhamnoside had the highest content at 12.68 min. Chlorogenic acid, rutin, isoquercitrin, quercetin-3-*O*-(6-*O*-malonyl)-β-d-glucoside, kaempferol 3-*O*-glucoside (astragalin), and kaempferol-3-*O*-(6-*O*-malonyl)-β-d-glucoside have previously been isolated from mulberry leaves of six different varieties (Lee and Choi 2012).

Quercetin have been reported many beneficial effects, such as anti-inflammatory, antihypertensive, vasodilator effects, antiobesity, antihypercholesterolemic and antiatherosclerotic activities (Sultana and Anwar 2008; Salvamani et al. 2014). In addition, kaempferol have been shown to augment the body’s antioxidant defense and reducing the risk of cancer (Chen and Chen 2013). However, the studies for biologically active of derivatives of quercetin and kaempferol in mulberry leaves are scarce, and it still remains to be seen whether these derivatives can really help augment biological effects in in-vivo of human. Thus, the 17 compounds including seven new compounds identified in mulberry leaves need that further investigation conducted to verify beneficial health effects for biologically active of human.

## Conclusion

Flavonoid components extracted from leaves of 12 mulberry varieties from Korean cultivars were quantified. UPLC–DAD–QTOF/MS analysis was used, and the mulberry leaves constituents were analyzed using complementary information obtained from LC spectra, MS ions, and MS/MS fragments. The seven of the 17 compounds identified were observed in mulberry leaves, and further research will be devoted for evaluating their biological activities. This information on the concentration of functional materials in mulberry leaves could contribute to the development and promotion of processed functional products and facilitate the possible industrial use of mulberry, which could enhance the overall profitability of sericulture.

## References

[CR1] Berardini N, Fezer R, Conrad J, Beifuss U, Carle R, Schieber A (2005). Screening of mango (*Mangifera indica* L.) cultivars for their contents of flavonol O- and xanthone C-glycosides, anthocyanins and pectin. J Agric Food Chem.

[CR2] Bharani SE, Asad M, Dhamanigi SS, Chandrakala GK (2010). Immunomodulatory activity of methanolic extract of *Morus alba* Linn. (mulberry) leaves. Pak J Pharm Sci.

[CR3] Braca A, Fico G, Morelli I, De Simone F, Tomè F, De Tommasi N (2003). Antioxidant and free radical scavenging activity of flavonol glycosides from different Aconitum species. J Ethnopharmacol.

[CR4] Chai OH, Lee MS, Han EH, Kim HT, Song CH (2005). Inhibitory effects of *Morus alba* on compound 48/80-induced anaphylactic reactions and anti-chicken gamma globulin IgE-mediated mast cell activation. Biol Pharm Bull.

[CR5] Chen AY, Chen YC (2013). Review: a review of the dietary flavonoid, kaempferol on human health and cancer chemoprevention. Food Chem.

[CR6] Chen PN, Chu SC, Chiou HL, Kuo WH, Chiang CL, Hsieh YS (2006). Mulberry anthocyanins, cyaniding 3-rutinoside and cyaniding 3-glucoside, exhibited an inhibitory effect on the migration and invasion of a human lung cancer cell line. Cancer Lett.

[CR7] Choi EM, Hwang JK (2005). Effects of *Morus alba* leaf extract on the production of nitric oxide, prostaglandin E2 and cytokines in RAW264.7 macrophages. Fitoterapia.

[CR8] Choi SW, Lee YJ, Ha SB, Jeon YH, Lee DH (2015). Evaluation of biological activity and analysis of functional constituents from different parts of mulberry (*Morus alba* L.) tree. J Korean Soc Food Sci Nutr.

[CR9] Enkhmaa B, Shiwaku K, Katsube T, Kitajima K, Anuurad E, Yamasaki M, Yamane Y (2005). Mulberry (*M. alba* L.) leaves and their major flavonol quercetin 3-(6-malonylglucoside) attenuate atherosclerotic lesion development in LDL receptor-deficient mice. J Nutr.

[CR10] Haminiuk CWI, Maciel GM, Plata-Oviedo MSV, Peralta RM (2012). Phenolic compounds in fruits—an overview. Int J Food Sci Technol.

[CR11] Jeong JH, Lee NK, Cho SH, Jeong YS (2014). Enhancement of 1-deoxynojirimycin content and α-glucosidase inhibitory activity in mulberry leaf using various fermenting microorganisms isolated from Korean traditional fermented food. Biotechnol Bioprocess Eng.

[CR12] Kalinova J, Triska J, Vrchotova N (2006). Distribution of vitamin E, squalene, epicatechin and rutin in common buckwheat plants (*Fagopyrum esculentum* Moech). J Agric Food Chem.

[CR13] Katsube T, Imawaka N, Kawano Y, Yamazaki Y, Shiwaku K, Yamane Y (2006). Antioxidant flavonol glycosides in mulberry (*Morus alba* L.) leaves isolated based on LDL antioxidant activity. Food Chem.

[CR15] Kicel A, Michel P, Owczarek A, Marchelak A, Zyzelewicz D, Budryn G, Oracz J, Olszewska MA (2016). Phenolic profile and antioxidant potential of leaves from selected *Cotoneaster* Medik. Species. Molecules.

[CR16] Kim HB, Kim AJ, Kim SY (2003). The study on the functional materials and effects of mulberry leaf. Food Sci Ind.

[CR17] Kim JW, Kim SU, Lee HS, Kim I, Ahn MY, Ryu KS (2003). Determination of 1-deoxynojirimycin in *Morus alba* L. leaves by derivatization with 9 fluorenylmethyl chloroformate followed by reversed-phase high-performance liquid chromatography. J Chromatogr A.

[CR18] Kim HW, Kim JB, Cho SM, Chung MN, Lee YM, Chu SM, Che JH, Kim SN, Kim SY, Cho YS, Kim JH, Park HJ, Lee DJ (2012). Anthocyanin changes in the Korean purple-fleshed sweet potato, Shinzami, as affected by steaming and baking. Food Chem.

[CR19] Kuti JO, Konuru HB (2004). Antioxidant capacity and phenolic content in leaf extracts of tree spinach (*Cnidoscolus* spp.). J Agric Food Chem.

[CR20] Lee WJ, Choi SW (2012). Quantitative changes of polyphenolic compounds in mulberry (*Morus alba* L.) leaves in relation to varieties, harvest period, and heat processing. Prev Nutr Food Sci.

[CR21] Lee JY, Moon SO, Kwon YJ, Lee SJ, Choi SW (2004). Identification and quantification of anthocyanins and flavonoids in mulberry (*Morus* sp.) cultivars. Food Sci Biotechnol.

[CR22] Materska M, Perucka I, Stochmal A, Piacente S, Oleszek W (2003). Quantitative and qualitative determination of flavonoids and phenolic acid derivatives from pericarp of hot pepper fruit cv. Bronowicka Ostra. Pol J Food Nutr Sci.

[CR23] Panda S, Kar A (2007). Annona squamosa leaves are possibly mediated through quercetin-3-O-glucoside. BioFactors.

[CR24] Salvamani S, Gunasekaran B, Shaharuddin NA, Ahmad SA, Shukor MY (2014). Antiartherosclerotic effects of plant flavonoids. Biomed Res Int.

[CR25] Singab AN, El–Beshbishy HA, Yonekawa M, Nomura T, Fukai T (2005). Hypoglycemic effect of Egyptian *Morus alba* root bark extract: effect on diabetes and lipid peroxidation of streptozotocin-induced diabetic rats. J Ethnopharmacol.

[CR26] Sultana B, Anwar F (2008). Flavonols (Kaempeferol, quercetin, myricetin) contents of selected fruits, vegetables and medicinal plants. Food Chem.

[CR27] Thabti I, Elfalleh W, Hannachi H, Ferchichi A, Da Graça Campos M (2012). Identification and quantification of phenolic acids and flavonol glycosides in Tunisian *Morus* species by HPLC–DAD and HPLC–MS. J Funct Foods.

[CR28] Thabti I, Elfalleh W, Tlili N, Ziadi M, Campos MG, Ferchichi A (2014). Phenols, flavonoids, and antioxidant and antibacterial activity of leaves and stem bark of *Morus* species. Int J Food Prop.

[CR29] Wang J, Wu FA, Zhao H, Liu L, Wu QS (2008). Isolation of flavonoids from mulberry (*Morus alba* L.) leaves with macroporous resins. Afr J Biotechnol.

[CR30] Wiczkowski W, Piskuła MK (2004). Food flavonoids. Pol J Food Nutr Sci.

[CR31] Zhang D, Wan Y, Xu J (2016). Ultrasound extraction of polysaccharides from mulberry leaves and their effect on enhancing antioxidant activity. Carbohydr Polym.

